# Effectiveness of exercise interventions on sperm quality: a systematic review and network meta-analysis

**DOI:** 10.3389/fendo.2025.1537271

**Published:** 2025-03-04

**Authors:** Weijia Song, Yu Peng, Zhuyu Jiang, Zheping Quan

**Affiliations:** ^1^ College of Education, Cavite State University, Cavite, Philippines; ^2^ College of Education, Manuel L. Quezon University, Quezon, Philippines; ^3^ College of Physical Education, Taiyuan Normal University, Jinzhong, Shanxi, China

**Keywords:** sports interventions, sperm quality, effectiveness, male infertility, systematic review, network meta-analysis

## Abstract

**Background:**

Infertility affects about 10% to 15% of the world’s population, thus making it a global concern. Although there are a large number of studies to develop treatment for infertility in men, there are no studies to illustrate the effect of exercise on male sperm treatment in a well rationalized and aggregated manner, therefore the aim of this study was to validate the comparative effectiveness of different exercise interventions for treating sperm quality in men by using a network Meta-analysis.

**Methods:**

All randomized clinical trials (RCT) were obtained from PubMed, Cochrane Library, EMBASE, Web of Science, CNKI, Wanfang, and VIP databases, and network meta-analysis was used to assess the effectiveness of exercise interventions on sperm quality.

**Results:**

Fourteen studies with 1079 subjects were finally included in this study. Compared with the physical inactivity group, indoor aerobic, outdoor aerobic, and resistance training significantly reduced sperm density (P<0.05); indoor aerobic, outdoor aerobic, and resistance training significantly reduced the number of necrotic spermatozoa and the number of active spermatozoa (P>0.05).Outdoor aerobic[MD=1.84,95%CI:(1.56,2.14),P<0.05], multi-component motion [MD=1.37,95%CI:(0.85,1.89),P<0.05],competitive sports[MD=1.04,95%CI:(0.46,1.60),P<0.05],indoor aerobic [MD=0.32, 95%CI:(0.21,0.44), P<0.05], effectively enhanced sperm volume; other sports [MD=9.49,95%CI:(6.17,12.84),P<0.05], indoor aerobic[MD=4.43,95%CI:(3.12,5.74),P<0.05],resistance training [MD=3.93, 95%CI:(0.49,7.37), P<0.05], competitive sports [MD=5.44,95%CI:(0.10,10.79), P<0.05], and bicycle aerobics[MD=27.29,95%CI:(22.45,32.06),P<0.05], significantly enhanced sperm motility; other sports [MD=17.20,95%CI:(3.12,31.19), P<0.05], effectively enhanced total sperm count;resistance training[MD=10.90,95%CI:(8.44,13.36), P<0.05],other sports [MD=1.97,95%CI:(1.41,2.54),P<0.05], indoor aerobic [MD=2.43,95%CI:(1.13,3.73), P<0.05],and bicycle aerobic [MD=12.18, 95%CI:(10.19,14.18),P<0.05], significantly enhanced sperm morphology; bicycle aerobic (MD=18.87, 95%CI:11.70,25.86, P<0.05), and indoor aerobic (MD=9.53, 95%CI:8.97,10.09, P<0.05),effectively enhanced sperm concentration.

**Conclusion:**

In conclusion, outdoor aerobics had a significant effect on improving sperm volume in infertile patients; other sports had a significant effect on enhancing sperm motility and total sperm count in infertile patients; resistance training had a significant effect on enhancing sperm morphology in infertile patients, and bicycle aerobic has a significant impact on improving sperm concentration in infertile patients.

**Systematic review registration:**

https://www.crd.york.ac.uk/prospero/#myprospero, identifier CRD42024534582.

## 1 Introduction

The World Health Organization(WHO)has declared infertility as a major global public health problem in the last decades (1). Infertility is defined as the absence of pregnancy after 12 months or more of appropriate and well-timed unprotected sexual intercourse (2). It is estimated that infertility affects about 10%to 15%of the world’s population, thus making it a global concern (3). Approximately 186 million people suffer from infertility worldwide, more than half of whom are male infertility (4). In recent years, an increasing number of studies have emphasized the influence of factors such as inflammation of the reproductive tract, irregular lifestyle, and nutritional deficiencies on the development of male infertility (5). In this regard, obesity and other conditions such as alcoholism, metabolic syndrome, smoking, and the environment are strongly associated with decreased sperm quality and fertility. Thus, sperm quality can influence ejaculatory competitiveness and conception success (6).

In addition, it has been reported that approximately 30-80%of male infertility is thought to be due to the negative effects of oxidative stress on spermatozoa (7). It occurs when reactive oxygen species(ROS)exceed the antioxidant defenses of semen, which damages proteins, DNA, and lipids (8). Oxidative stress-induced sperm DNA damage can lead to decreased sperm viability, acrosomal membrane damage, decreased sperm fertilization, and ultimately decreased fertility (9). Therefore, effective interventions to improve male reproductive function are imminent. Currently, the main drugs used in the clinical treatment of male infertility are antioxidants, hormones, hexacosanolone cocaine, and levocarnitine (LC) (10), which have the drawbacks of uncontrolled side effects, expensive price, case dependence, and poor prognosis. It has been suggested (11) that a healthy lifestyle such as regular exercise, sensible diet, and smoking cessation may bring about a more favorable environment for reproduction-related processes and can improve or prevent the regression of hormonal and semen parameters. Aerobic exercise, as one of the important lifestyles for health promotion, not only relieves psychological stress and improves sleep quality, but also improves the adaptability of muscle and cardiorespiratory functions, enhances the body’s ability to fight against stress responses, and delays the clinical symptoms of some chronic diseases (12). At the same time, exercise and non-pharmacological interventions such as acupuncture, massage and gua sha have been developed and applied in the management and treatment of male infertility (13). Non-pharmacological treatments, especially through exercise intervention modalities, exercise intervention efficacy is controllable and has no side effects, so it can be used to improve sperm quality, vitality, and morphology through interventions such as changing exercise modalities, thus improving the overall fitness of men (14).

From the available research results, although studies have been conducted to develop treatments for infertility in men, due to the many causes of oligospermia, many of the mechanisms have not been fully defined and treatment outcomes vary from person to person. Previous systematic reviews have analyzed the efficacy and safety of different treatments for male infertility, but have not yet compared the effects of different exercise modality interventions. Therefore, the aim of our study was to validate the comparative effectiveness of different exercise interventions for treating male sperm quality by reticulated Meta-analysis, in order to seek for therapies with significant efficacy, stable effects and safety.

## 2 Materials and methods

### 2.1 Registration

The study protocol has been registered on the International Prospective Systematic Evaluation Registry (PROSPERO) platform under the registration number CRD42024534582 (15).

### 2.2 Search strategy

The system searched four English databases, PubMed, Cochrane Library, EMBASE, and Web of Science, and three Chinese databases, including CNKI, Wanfang, and VIP, with the year of publication of the articles up to March 2024 from the time of database construction. The following MESH terms were applied to search for relevant literature (the search strategy was based on PubMed as an example): (“exercise”[MeSH Terms] OR “exercise”[All Fields] OR “exercises”[All Fields] OR “exercise therapy”[MeSH Terms] OR (“exercise”[All Fields] AND “therapy”[All Fields]) OR “exercise therapy”[All Fields] OR “exercise s”[All Fields] OR “exercised”[All Fields] OR “exerciser”[All Fields] OR “exercisers”[All Fields] OR “exercising”[All Fields]) AND (“intervention s”[All Fields] OR “interventions”[All Fields] OR “interventive”[All Fields] OR “methods”[MeSH Terms] OR “methods”[All Fields] OR “intervention”[All Fields] OR “interventional”[All Fields]) AND ((“sperm s”[All Fields] OR “spermatozoa”[MeSH Terms] OR “spermatozoa”[All Fields] OR “sperm”[All Fields] OR “sperms”[All Fields]) AND (“qualities”[All Fields] OR “quality”[All Fields] OR “quality s”[All Fields]))。

### 2.3 Selection criteria

#### 2.3.1 Inclusion criteria

Inclusion criteria for the article were determined using the PICOS (Participants/Interventions/Comparisons/Outcomes/Research Design) principle as follows. Participants (P): Normal or non-congenitally infertile males without thyroid disease, chronic disease, etc., and the age range or average age of the subjects was between 18 and 60 years. Intervention (I): The experimental group adds an exercise intervention to the control group, where the type of exercise is one sport or a combination of more than one sport and is not combined with other interventions, etc. Comparison (C): Routine intervention, sedentary or no sports intervention, etc., all of the above were without any regular physical activity. Outcome (O). sperm volume, sperm concentration, sperm motility, sperm morphology, total sperm count, sperm density, Number of active spermatozoa, number of necrotic spermatozoa. Research Design: Clinically relevant randomized controlled trial with publication year from inception to March 2024.

#### 2.3.2 Exclusion criteria

(1) The study was conducted in men with thyroid disease, chronic disease, or congenital infertility; (2) Review literature, descriptive literature, conferences; (3) Articles not published in English or Chinese; (4) Articles with the same data as other included studies; (5) Literature with incomplete outcome index data, resulting in data that cannot be extracted; (6) Infertility due to female factors; (7) Infertility caused by obstruction, hypothalamic-pituitary lesions, congenital anomalies, and endogenous or exogenous hormonal abnormalities that are not caused by traumatic brain injury, space-occupying lesions, or other neurologic disorders.

### 2.4 Data extraction

In the included studies, two investigators (WS and YP) independently extracted data information from the same studies and then met to review their results and cross-check them, and any disagreements should be resolved by consensus. If consensus could not be reached, a third scholar (ZJ) provided suggestions for missing information in the text by contacting the original authors by email. After careful study of the title and abstract and elimination of irrelevant literature, literature that passed the initial screening should be reviewed in depth in the full text to clarify whether it is necessary to include it. Data items for data extraction were as follows: 1) first author and year of publication; 2) sample size, subjects (age, gender); 3) interventions and controls, duration of interventions, frequency of interventions, and cycle of interventions; and 4) outcome indicators as well as any differences in scores before and after interventions for each group.

### 2.5 Assessment of risk of bias

Two researchers (WS and PQ) independently evaluated and validated the quality of the literature using the Cochrane 2.0 Handbook Randomized Controlled Trial Risk of Bias Scale (16). The evaluation included seven entries: randomized sequence generation, allocation concealment, blinding of participants and investigators, blinding of outcome measures, completeness of outcome metrics, selective reporting, and other biases, each of which included “high risk”, “low risk”, “unclear”, “high risk”, “low risk”, “low risk”, and “unclear”. “unclear” (17). A schematic diagram of the literature risk of bias assessment was produced using R4.3.3 software.

### 2.6 Statistical analysis

Revman 5.4 and R 4.3.3 software were used for Pairwise Meta and network Meta-analysis of the data. The outcome indicators required for this study were all continuous variables, and because mean difference is an indicator that directly quantifies the mean difference between different treatment or control groups, and when the purpose of the study is to compare the means of two or more groups, the mean difference can clearly and intuitively reflect the degree of difference between the groups, therefore, this study used mean difference (MD) and its 95% confidence interval (confidence interval, CI) were used as the effect size indicators in this study.

Firstly, the heterogeneity (I^2^) and P-value of different exercise modalities in direct comparison with the control group were obtained by Pairwise Meta-analysis, and sensitivity or subgroup analyses could be done if necessary. Secondly, network Meta-analysis was performed to draw network relationship diagrams, league diagrams, and cumulative probability ranking diagrams. Network evidence maps in the network Meta-analysis visualize the relationship between direct and indirect comparisons between the various exercise interventions. Through the convergence diagnosis of the five outcome indicators, in the convergence diagnosis, the fixed-effects model based on the Bayesian school was constructed by the Markov chain-Monte Carlo method, and the constructed model was iterated to achieve a satisfactory degree of convergence, and the model convergence results were more satisfactory in this study when the potential scale reduction factor (PSRF) was between 1 and 1.05. In this study, when the potential scale reduction factor (PSRF) is in the range of 1 to 1.05, it suggests that the model convergence results are satisfactory. When the Network graph formed a closed loop, the inconsistency test was conducted using the node splitting method to determine the difference between the direct comparison results and the indirect comparison results, if P>0.05 suggests that the two results are consistent; the effect ranking of each exercise intervention will be combined with the amount of the effect and the value of the SUCRA for the interpretation, the larger the value of the SUCRA indicates that the possibility of the method to become the optimal intervention method is greater. Finally, funnel plots were drawn using R software to assess whether there was publication bias in the intervention.

## 3 Results

### 3.1 Literature search results

**Figure 1:**
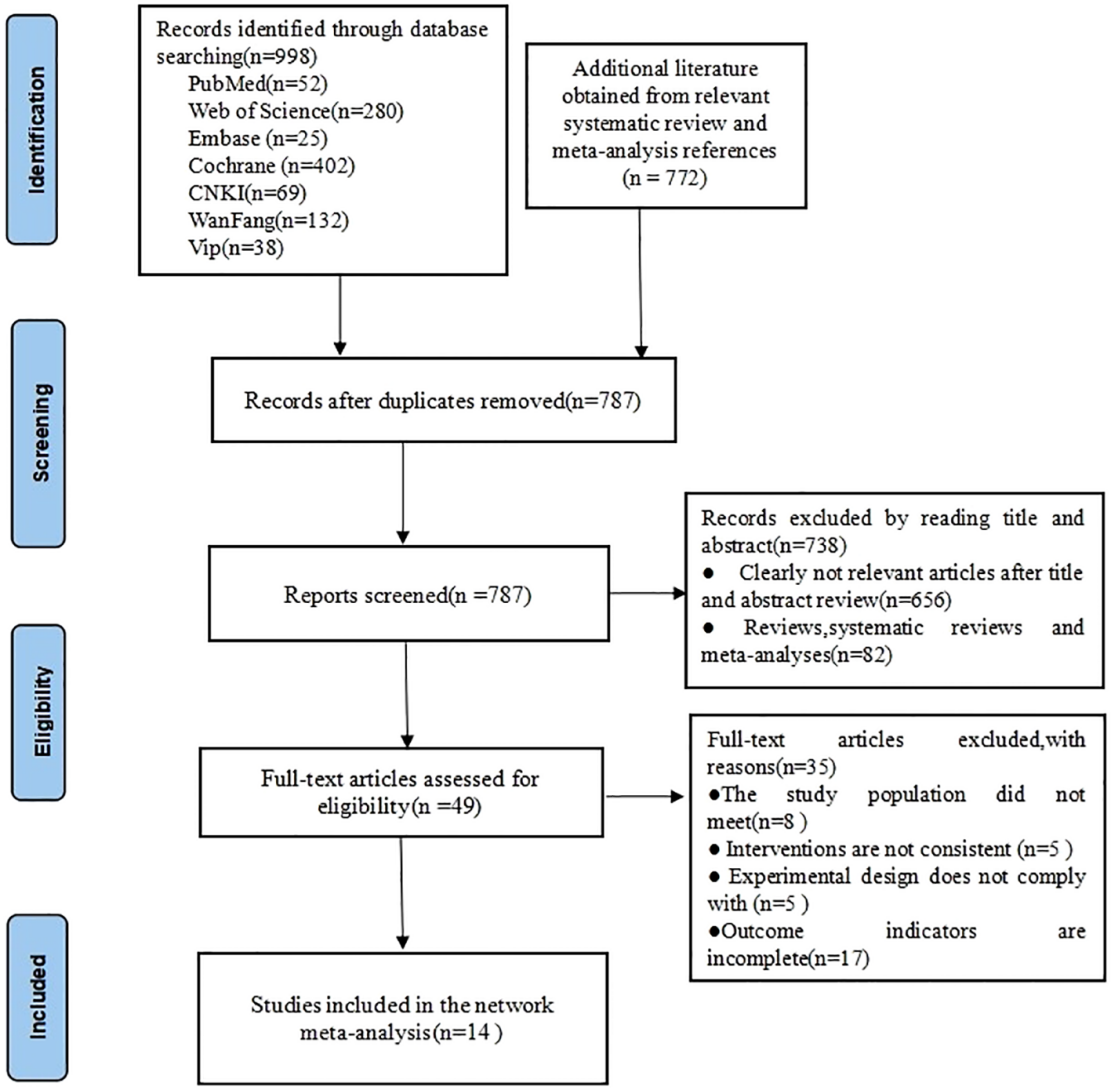
Literature search flow chart.

In this study, a total of 1,770 pieces of related literature were retrieved, including 38 pieces of VIP, 69 pieces of China Knowledge Network, 132 pieces of Wanfang, 52 pieces of PubMed, 25 pieces of Embase, 280 pieces of Web of Science, 402 pieces of Cochrane, and 772 pieces of literature obtained from other resources, and 787 pieces of literature were left over after elimination of duplicates, and the titles and abstracts were read (initial screening)Excluding literature 738, read the full-text re-screening of the randomized controlled literature 49, according to the study population, interventions, trial design and outcome indicators incomplete exclusion of literature 35, the final inclusion of literature 14, see **Figure 1**.

### 3.2 Basic characteristics of included studies

A total of 17 independent samples from 14 papers were included, which were published from 1989 to 2022, with a total of 1079 subjects in the experimental and control groups, 610 in the experimental group, and 469 in the control group. The main interventions utilized in the experimental group were eight types of outdoor aerobic, indoor aerobic, multi-component motion, competitive sports, resistance training, bicycle aerobic, aerobic endurance exercise, and other sports. In contrast, the control group mainly did not engage in any regular physical activity. The basic characteristics of the included literature are shown in **Table 1**.

**Table 1:** Basic characteristics of the included literature.

Study	Year	Interventions	Sample size	Age	Measure time	Outcomes
Rosety (18)	2013	IA	30	36.2 ± 3.5	40min/time, 3 times/week, 14 weeks	①⑤③④
		NE	30	35.7 ± 4.0	—	
Rafiee A (19)	2016	IA	50	20-60	24 weeks	①②
		NE	50	20-60	—	
Rafiee B (19)	2016	IA	50	20-60	24 weeks	①②
		NE	50	20-60	—	
Rafiee C (19)	2016	IA	50	20-60	24 weeks	①②
		NE	50	20-60	—	
Denham (20)	2015	IA	13	24.4 ± 5.19	1time/2week,12weeks	①⑦
		NE	11	22.45 ± 4.74	—–	
Vaamonde (21)	2009	MM	16	19.0 ± 1.8	60min/time,3time/week	①②⑤
		CS	14	25.5 ± 3.2	90min/time,5time/week	
		OA	15	33.1 ± 3.5	32km/day, No show	
Ali Mohamed (22)	2023	IA	20	33.60 ± 4.14	40min/time,3times/week, 14 weeks	①③④⑤
		NE	20	34.05 ± 4	No show,14weeks	
Montano (23)	2021	OS	137	19.3 ± 1.4	1times/week, 16 weeks	①②③④
		NE	126	19.3 ± 1.4	No show,16 weeks	
Arce (24)	1993	RT	8	24.6 ± 1.8	120min/day, 4days/week, 12month	①③④⑤⑥
		OA	10	27.6 ± 1.9	96km/week,12month	
		NE	10	29.2 ± 1.7	<1h/week,12month	
B.Hajizadeh Maleki (25)	2012	BA	56	23.8 ± 5.2	2h/time,4-5time/week, 6month	①②③④⑤
		OS	52	24.2 ± 4.9	4-5h/time, 2-3day/week,6month	
		NE	53	23.9 ± 5.0	No show,6month	
M. F. CELANI (26)	1989	CS	10	22.2 ± 2.2	90min/week,3month	①③⑤
		MM	10	22.2 ± 2.2	2times/week, 3month	
		OS	10	22.2 ± 2.2	No show,30day	
		NE	10	22.6 ± 2.4	No show	
M. J De Souza A (27)	1994	IA	11	27.6 ± 1.7	104km/h,12month	①④⑤⑥⑦⑧
		NE	10	29.2 ± 1.7	<1h/week,No show	
M. J De Souza B (27)	1994	IA	9	28.1 ± 1.3	40-56km/h,12month	①④⑤⑥⑦⑧
		NE	10	29.2 ± 1.7	<1h/week,No show	
Vaamonde (28)	2012	AEE	16	19.2 ± 1.9	2-4h/week, 3day	①②
		NE	15	19.0 ± 1.8	No show	
Xiaolin Zhang (29)	2019	OA	3	19	50min/time,6time/week,6weeks	③⑦
		NE	4	19	No show	
Carrie J (30).	1990	OA	11	32.7 ± 1.2	40km/week,No show	③⑤
		NE	12	32.2 ± 1.4	No show	
Andersen (31)	2022	OA	9	37 (range 19–60)	30min/time,5time/week,52weeks	①②③⑤⑦
		NE	8	37 (range 19–60)	No show,52weeks	

Outdoor Aerobic: Includes running,biking,swimming, hiking, and other exercises that can be done outdoors. Indoor Aerobic: Examples of exercise that can be done in a gym or at home such as treadmill,kinetic cycling, jumping rope and aerobics. Multi-component motion: a combination of strength training, flexibility training, aerobic exercise and coordination training. Competitive sports: include sports such as soccer, basketball, tennis, etc. that require confrontation or competition. Resistance Training: refers to training that increases muscular strength and endurance through weight bearing, such as weight lifting, machine training, and elastic band training. Bicycle aerobics: Includes outdoor cycling and indoor dynamic cycling, mainly at low to moderate intensity for continuous exercise. Aerobic Endurance Exercise: Aerobic exercise performed at lower intensity for a long period of time, such as long-distance running, long-distance swimming and long-distance cycling. Other Sports: This category includes activities that are not easily categorized or that combine multiple forms of exercise, such as yoga, Pilates, tai chi, dance, and recreational activities. ①Sperm volume②Sperm concentration③sperm motility④Sperm morphology⑤Total sperm count⑥Sperm density⑦Number of active spermatozoa⑧Number of necrotic spermatozoa; OA, Outdoor Aerobic; IA, Indoor Aerobic; MM, Multi-component motion; CS, competitive sports; OS, other sports; RT, resistance training; BA, Bicycle aerobics; AEE, aerobic endurance exercise; NE, No Exercise.”—”: Not involved.

### 3.3 Risk of bias assessment

**Figure 2:**
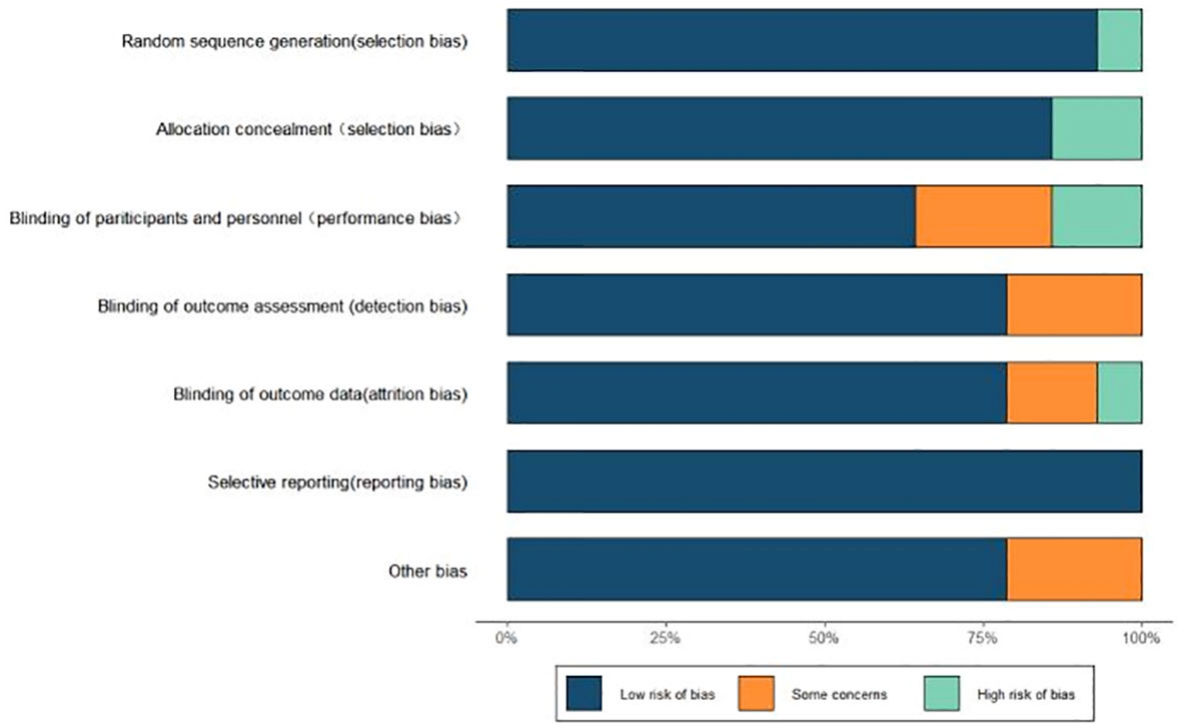
Results of risk of bias evaluation of included studies.

Of the 14 papers included, 13 papers reported the random allocation method as random number table method/computer random generation, and the remaining one did not detail the allocation method; 13 papers performed allocation concealment; 9 papers were blinded to the intervener or outcome measure in the study, the rest were not detailed; 11 papers reported the number of dropouts and the reasons for them; 11 studies had no residuals in the outcome data, and all papers did not selectively report results (see **Figure 2**).

### 3.4 Pairwise meta-analysis

Categorical analysis by indicators Due to the small number of literature on the number of active spermatozoa, the number of necrotic spermatozoa, and sperm density included in the study for the three indicators, only the experimental group was compared with the control group, which did not have the conditions for network Meta-analysis, so only Pairwise Meta-analysis was performed (see **Table 2**).

**Table 2:** Results of Pairwise meta-analysis.

Outcome	Heterogeneity test		Effect model	Meta-analysis		Subgroup differences(P)
	I^2^%	P value		MD and 95% CI	P value	
Number of active spermatozoa	91	<0.00001	RE	-12.16(-39.52,15.19)	0.38	
OA	93	<0.00001	RE	-18.43(-67.79,30.92)	0.46	0.30
IA	91	<0.00001	RE	8.00(-1.03,17.03)	0.08	
Number of necrotic spermatozoa	95	<0.0001	RE	-38.23(-141.74,65.24)	0.47	×
OA	RE					
Sperm density	83	0.0006	RE	-72.86(-96.36,-49.36)	<0.00001	
OA	×	×	RE	-98.00(-115.19,-80.81)	<0.00001	0.003
IA	83	0.02	RE	-68.88(-106.73,-31.03)	0.0004	
RT	×	×	RE	-54.00(-72.66, -35.34)	<0.00001

RE, random effects model; OA, Outdoor Aerobics; IA, Indoor Aerobics; RT, resistance training; “×” indicates not available.

#### 3.4.1 Number of active spermatozoa

Four studies included continuous changes in the number of active spermatozoa after the intervention. Pairwise Meta-analysis showed a large heterogeneity with I^2^ = 91%, so the RE model was used. The results of the combined effect showed that [MD=-12.16,95%CI:(-39.52,15.19),Z=0.87,P>0.05]. The study showed that there was no statistically significant difference in the improvement of men’s number of active spermatozoa with exercise compared to no exercise, and no significant effect was seen. Analysis based on the interventions showed that there was high heterogeneity in the intervention for indoor aerobic (I^2^ = 93%,P<0.00001), and there was no difference between the experimental group and the control group after the intervention, and the results were not statistically significant [MD=-18.43,95%CI:(-67.79,30.92),P=0.46]; and there was high heterogeneity in the intervention for outdoor aerobic(I^2^ = 91%,P<0.00001),there was no difference between the experimental and control groups after the intervention, and the results were not statistically significant[MD=8.00,95%CI:(-1.03,17.03),P=0.08].There was no difference in the comparison of the Number of active spermatozoa by different exercise interventions(P=0.30),and it could not be shown that different exercises were a source of heterogeneity in the results affecting active sperm counts.

#### 3.4.2 Number of necrotic spermatozoa

One study included serial changes in necrotic sperm count after intervention. Pairwise Meta-analysis showed a large heterogeneity with I^2^ = 95%, so the RE model was used. The results of the combined effect showed that[MD=-38.23,95%CI:(-141.74,65.24),Z=0.72,P>0.05].The study showed that there was no statistically significant difference in the improvement of the Number of necrotic spermatozoa.by exercise compared to the no exercise group and no significant effect was seen.

#### 3.4.3 Sperm density

Three studies listed continuous changes in sperm density after intervention. Pairwise Meta-analysis showed a large heterogeneity with I^2^ = 95%, so the RE model was used. The results of the combined effect showed that [MD=-72.86,95%CI:(-96.36,-49.36),Z=6.08,P<0.05].The study showed a statistically significant effect of exercise on improving sperm density compared to no exercise. The analysis based on the interventions showed that: the intervention for indoor aerobics reduced the sperm density of the subjects after the intervention, and the result was statistically significant[MD=-98.0,95%CI:(-115.19,-80.81),P<0.00001]; there was a high degree of heterogeneity in the intervention for outdoor aerobic (I^2^ = 83%,P=0.02),and the sperm density of the experimental group was lower than the control group after the intervention, and the result was statistically significant [MD=-68.88,95%CI:(-106.73,-31.03),P=0.0004];the intervention for resistance training showed that Sperm density was lower than the control group, and the result was statistically significant[MD=-68.88,95%CI:(-106.73,-31.03),P=0.0004]; the intervention was resistance training intervention reduced the sperm density of the subjects, and the result was statistically significant [MD=-54.00,95%CI:(-72.66,-35.34),P<0.00001].There was a difference in the comparison of the Number of active spermatozoa by different exercise interventions (P=0.003),suggesting that different exercise types are a source of heterogeneity in the results affecting sperm density.

#### 3.4.4 Sensitivity analysis

Sensitivity analyses were performed by eliminating sources of heterogeneity, and due to differences in the heterogeneity of the included outcome indicators, data were analyzed using different effects models to determine the stability of the results of this study. The results of the effect sizes obtained from the transformed effects model were compared, and the combined effect sizes of the risk factors were close to each other without differential changes, indicating that the results of the Meta-analysis were stable, and vice versa and analyzing the factors of instability.

The results of the sensitivity test showed(see **Table 3**): that by converting the values of the combined effect sizes of the fixed-effects model and the random-effects model for comparison, the combined effect sizes of the risk factors were close to each other, and it was found that the fixed-effects model was in the confidence interval of the random-effects model, and there was no differential change, and the results of the Meta-analysis were more stable, with low sensitivity and good stability.

**Table 3:** Pairwise meta sensitivity analysis.

Outcome	Effect model	Effect size	95% confidence interval	Effect model	Effect size	95% confidence interval
Number of active spermatozoa	FE	MD= 2.58	[-4.02,9.19]	RE	MD=-12.16	[-39.52, 15.19]
OA	FE	MD=-3.67	[-13.37,6.03]	RE	MD=-18.43	[-67.79, 30.92]
IA	FE	MD=8.00	[-1.03,17.03]	RE	MD=8.00	[-1.03, 17.03]
Number of necrotic spermatozoa	FE	MD=-53.27	[-75.89,-30.65]	RE	MD=-38.23	[-141.71, 65.24]
OA	FE	MD=-53.27	[-75.89, -30.65]	RE	MD=-38.23	[-141.71, 65.24]
Sperm density	FE	MD=-76.51	[-86.08,-66.93]	RE	MD=-72.86	[-96.36,-49.36]
OA	FE	MD=-98.00	[-115.19,-80.81]	RE	MD=-98.00	[-115.19,-80.81]
IA	FE	MD=-74.77	[-89.44, -60.10]	RE	MD=-68.88	[-106.73,-31.03]
RT	FE	MD=-54.00	[-72.66, -35.34]	RE	MD=-54.00	[-72.66, -35.34]

RE, random effects model; FE, fixed effects model; OA, Outdoor Aerobics; IA, Indoor Aerobics; RT, resistance training; Resistance Training.

### 3.5 Network meta-analysis

#### 3.5.1 Network relationship diagram

**Figure 3:**
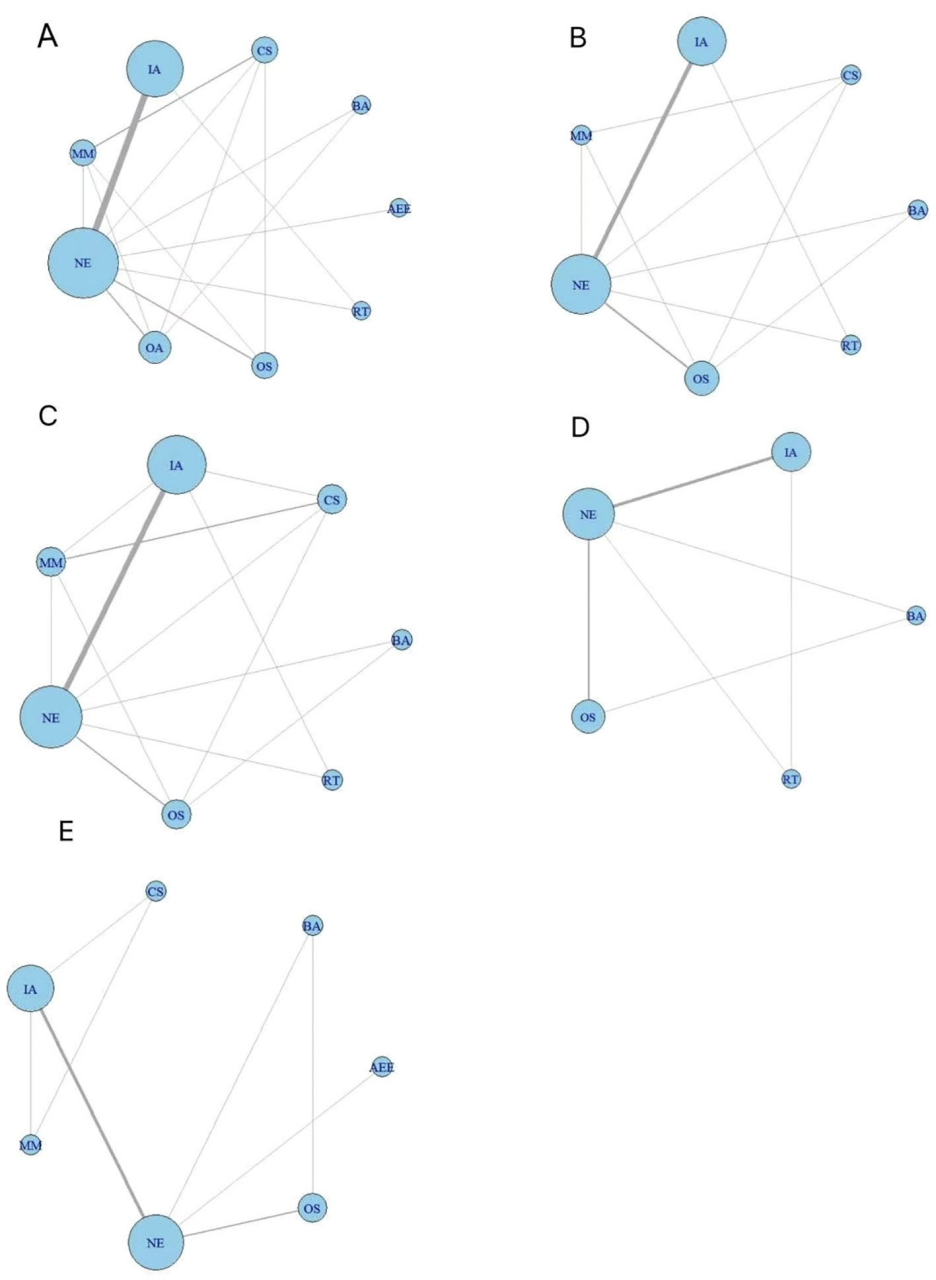
Diagram of the network of evidence across exercise interventions. **(A)** sperm volume; **(B)** sperm motility; **(C)** total sperm count; **(D)** sperm morphology; **(E)** sperm concentration. OA, Outdoor Aerobic; IA, Indoor Aerobic; MM, Multi-component motion; CS, competitive sports; OS, other sports; RT, resistance training; BA, Bicycle aerobics; AEE, aerobic endurance exercise; NE, No Exercise.

Using R software to network reticulations(see **Figure 3**),fifteen studies reported sperm volume metrics across nine exercise interventions and non-exercise measures, resulting in eleven 2-arm studies, three 3-arm studies, and one 4-arm study. Nine studies reported sperm motility indicators involving seven exercise interventions and non-exercise measures, resulting in six 2-arm studies, two 3-arm studies, and one 4-arm study. Ten studies reported total sperm count metrics involving seven exercise interventions as well as non-exercise measures, resulting in six 2-arm studies, three 3-arm studies, and one 4-arm study. Sperm morphology indicators were reported in seven studies involving five exercise interventions and non-exercise measures, resulting in five 2-arm studies and two 3-arm studies. Sperm concentration indicators were reported in eight studies involving seven exercise interventions and non-exercise measures, resulting in six 2-arm studies and two 3-arm studies.

#### 3.5.2 Sperm volume

**Figure 4:**
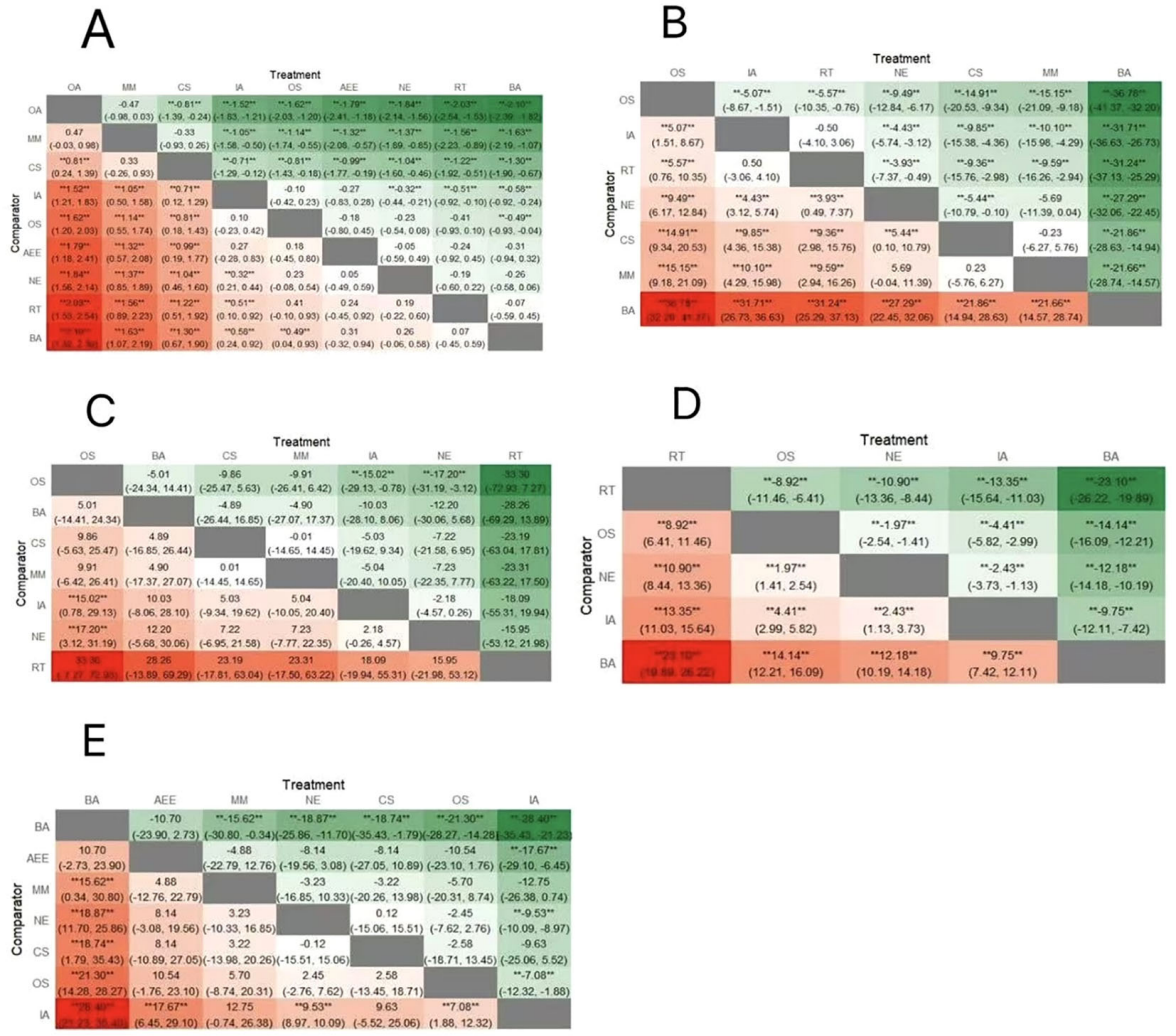
Network meta-analysis results. OA, Outdoor Aerobics; IA, Indoor Aerobics; MM, Multi-component motion; CS, competitive sports; OS, other sports; RT, resistance training; BA, Bicycle aerobics; AEE, aerobic endurance exercise; NE, no-exercise. **(A)** sperm volume; **(B)** sperm motility; **(C)** total sperm count; **(D)** sperm morphology; **(E)** sperm concentration.

A network meta-analysis of the included studies produced 36 two-by-two comparisons. As shown in **Figure 4**, a total of 15 studies reported on sperm volume with a sample size of 1119.Network Meta-analysis showed that outdoor aerobic [MD=1.84,95%CI:(1.56,2.14),P<0.05],multi-component sports [MD=1.37,95%CI:(0.85,1.89),P<0.05],competitive sports[MD=1.04,95%CI:(0.46,1.60),P<0.05],and indoor aerobic[MD=0.32,95%CI:(0.21,0.44),P<0.05],indicating that the effects of outdoor aerobic, multi-component movement, competitive sports, and indoor aerobic were significantly better than those of the no-exercise group in terms of enhancing sperm volume. In addition, other sports [MD=0.23,95%CI:(-0.08,0.54), P>0.05] and aerobic endurance exercise [MD=0.05, 95%CI: (-0.49,0.59), P>0.05], resistance training[MD=0.19,95%CI:(-0.22,0.60), P>0.05],bicycle aerobic[MD=0.26,95%CI:(-0.06,0.58),P>0.05], with no statistically significant difference in the results compared to the no-exercise group. A two-by-two comparison showed that outdoor aerobic was superior to competitive sports [MD=0.81,95%CI:(0.24,1.39),P<0.05], indoor aerobic[MD=1.52, 95%CI:(1.21,1.83),P<0.05], other sports [MD=1.62,95%CI:(1.20,2.03),P<0.05], aerobic endurance exercise [MD=1.79,95%CI:(1.18,2.41),P<0.05], resistance exercise[MD=2.03,95%CI:(1.53,2.54), P<0.05], and Bicycle aerobics [MD=2.10, 95%CI:(1.82,2.39), P<0.05]; multi-component motion was superior to indoor aerobic[MD=1.05, 95%CI:(0.50,1.58),P<0.05], other exercise [MD=1.14,95%CI: (0.55,1.74),P<0.05], aerobic endurance exercise [MD=1.32,95%CI:(0.57,2.08),P<0.05],resistance training[MD=1.56,95%CI:(0.89,2.23), P<0.05], Bicycle aerobics [MD=1.63,95%CI:(1.07,2.19), P<0.05];competitive sports was superior to indoor aerobics [MD=0.71,95%CI:(0.12,1.29),P<0.05],and other sports [MD=0.81,95%CI:(0.18,1.43),P<0.05],aerobic endurance exercise [MD=0.99,95%CI:(0.19,1.77),P<0.05],resistance training[MD=1.22,95%CI:(0.51,1.92), P<0.05],bicycle aerobic[MD=1.30,95%CI: (0.67,1.90), p<0.05];indoor aerobic was superior to resistance training[MD=0.51,95%CI:(0.10,0.92), p<0.05],and bicycle aerobics [MD=0.58,95%CI:(0.24,0.92),p<0.05]; other sports were superior to bicycle aerobics [MD=0.49, 95%CI:(0.04,0.93),P<0.05],and the rest of the differences between two-by-two comparisons with each other were not statistically significant(P>0.05).

**Figure 5:**
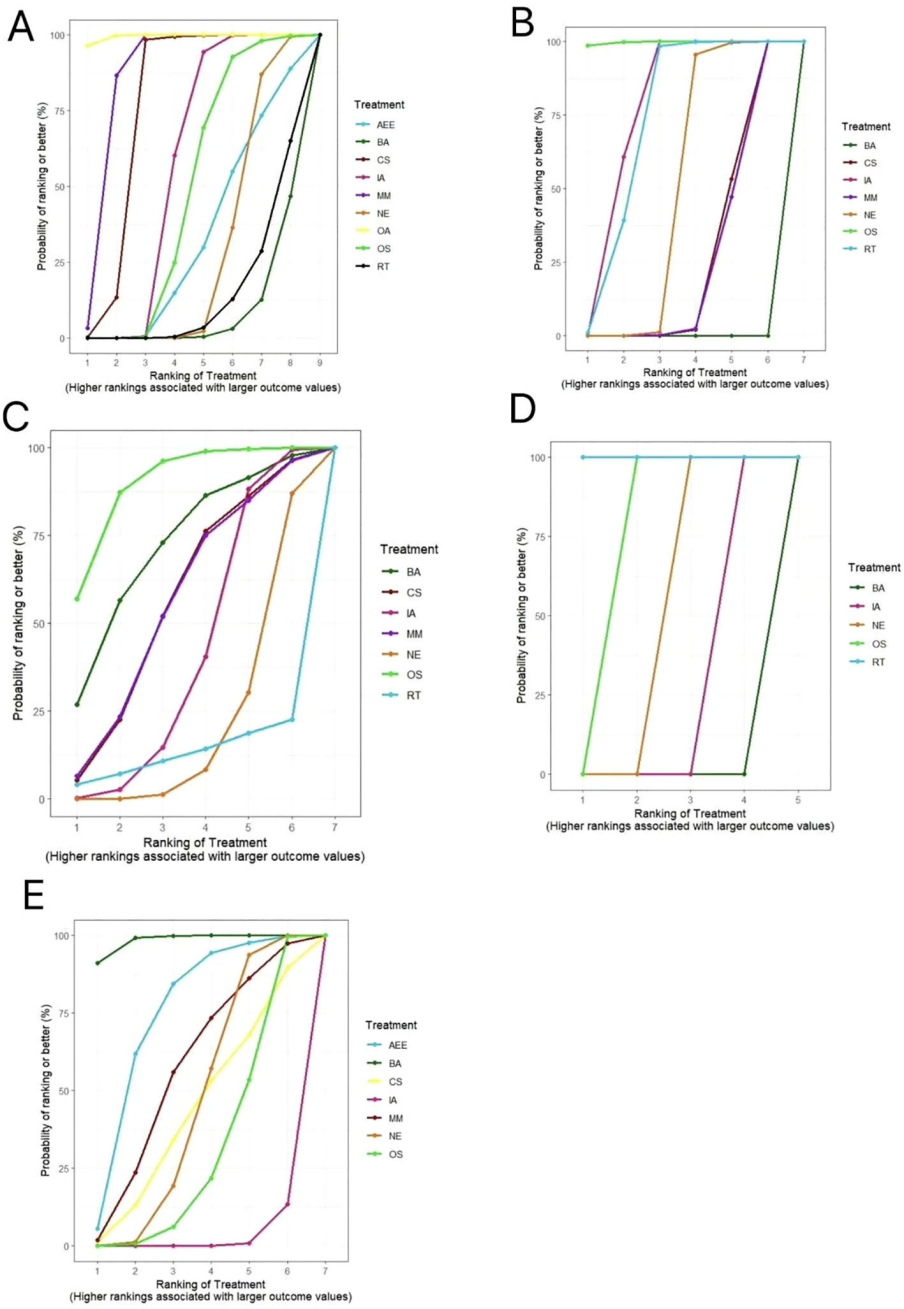
Area under the cumulative probability ranking curve. OA, Outdoor Aerobics; IA, Indoor Aerobics; MM, Multi-component motion; CS, competitive sports; OS, other sports; RT, resistance training; BA, Bicycle aerobics; AEE, aerobic endurance exercise; NE, no-exercise. **(A)** sperm volume; **(B)** sperm motility; **(C)** total sperm count; **(D)** sperm morphology; **(E)** sperm concentration.

The results of the SUCRA probability ranking showed that outdoor aerobic(SUCRA=99.21)> multi-component motion(SUCRA=86.16)>competitive sports(SUCRA=76.55)>indoor aerobic (SUCRA=57.10)>other sports (SUCRA=48.06) > aerobic endurance exercise (SUCRA=33.71)> no exercise (SUCRA=28.74) > resistance training (SUCRA=15.32)> bicycle aerobic(SUCRA=5.14),and outdoor aerobic was most likely to be the best intervention to improve sperm volume(see **Figure 5**).

#### 3.5.3 Sperm motility

A network meta-analysis of the included studies produced 21 two-by-two comparisons. As shown in **Figure 4**, a total of 9 studies reported on sperm motility with a sample size of 709. The results of network Meta-analysis showed that other sports [MD=9.49,95%CI:(6.17,12.84), P<0.05],indoor aerobic [MD=4.43,95%CI:(3.12,5.74), P<0.05],resistance training [MD=3.93,95%CI:(0.49,7.37), P<0.05],competitive sports[MD=5.44,95%CI:(0.10,10.79),P<0.05], bicycle aerobic [MD=27.29,95%CI:(22.45,32.06), P<0.05], indicating that there was a statistically significant difference in the efficacy of the intervention of the above five exercise modalities in terms of improvement of sperm viability as compared to that of the no exercise group difference. In addition, multi-component motion [MD=5.69, 95%CI: (-0.04,11.39), P<0.05], was not statistically different from the no-exercise group. A two-by-two comparison showed that other sports were superior to indoor aerobic[MD=5.07,95%CI:(1.51,8.67),p<0.05],resistance training[MD=5.57,95%CI:(0.76,10.35),p<0.05],competitive sports [MD=14.91,95%CI:(9.34,20.53), p<0.05],multi-component motion[MD=15.15,95%CI:(9.18,21.09), P<0.05], bicycle aerobics [MD=36.78,95%CI: (32.20,41.37), P<0.05]; and indoor aerobics was superior to competitive sports[MD=9.85,95%CI:(4.36,15.38),P<0.05],multi-component motion[MD=10.10,95%CI:(4.29,15.98),P<0.05],bicycle aerobic[MD=31.71,95%CI:(26.73,36.63),P<0.05];and resistance training was superior to competitive sports [MD=9.36,95%CI:(2.98,15.76),P<0.05],multi-component motion [MD=9.59,95%CI:(2.94,16.26), P<0.05], and bicycle aerobics [MD=31.24,95%CI:(25.29,37.13), P<0.05]; and competitive sports was superior to bicycle aerobics [MD=21.86,95%CI:(14.94,28.63),P<0.05]; multi-component motion was superior to bicycle aerobics[MD=21.66,95%CI:(14.57,28.74), P<0.05], and the difference between the other two comparisons was not statistically significant(P>0.05).

According to the results of SUCRA probability ranking, other sports (SUCRA=99.76) > indoor aerobic (SUCRA=76.83)> resistance training (SUCRA=73.09)> no exercise (SUCRA=49.31)>competitive sports (SUCRA=25.99)>multi-component motion (SUCRA=25.01) > bicycle aerobic (SUCRA=0.01), and other sports were most likely to be the best interventions for improving sperm motility (see **Figure 5**).

#### 3.5.4 Total sperm count

A network meta-analysis of the included studies produced 21 two-by-two comparisons. As shown in **Figure 4**, a total of 10 studies reported on total sperm counts with a sample size of 443. Network Meta-analysis showed that other sports [MD=17.20,95%CI:(3.12,31.19), P<0.05], indicating that other sports were significantly more effective than the non-exercise group in improving total sperm count. Bicycle aerobic[MD=12.20,95%CI:(-5.68,30.06),P>0.05],competitive sports [MD=7.22,95%CI:(-6.95,21.58),P>0.05],multi-component motion [MD=7.23, 95%CI:(-7.77,22.35), P>0.05], indoor aerobic[MD=2.18,95%CI:(-0.26,4.57), P>0.05], and resistance training [MD=-15.95, 95%CI:(-53.12,21.98), P>0.05], all of which did not show statistically significant differences in the results when compared to no exercise. Two-by-two comparison showed that other sports were superior to indoor aerobic [MD=15.02, 95%CI:(0.78,29.13), P<0.05], and there was no statistically significant difference in two-by-two comparison between the rest of the exercises with each other (P>0.05).

According to the results of SUCRA probability ranking, other sports (SUCRA=89.92)>bicycle aerobic exercise (SUCRA=71.35)>competitive sports(SUCRA=56.47)>multi-component motion(SUCRA=56.37)>indoor aerobic(SUCRA=41.16) >no exercise(SUCRA=21.36)>resistance training(SUCRA=13.37),and other sports were most likely to be the best interventions to improve sperm count (see **Figure 5**).

#### 3.5.5 Sperm morphology

A network meta-analysis of the included studies produced 10 two-by-two comparisons. As shown in **Figure 4**, 7 studies reported on sperm morphology, with a sample size of 977. The results of the network Meta-analysis showed that resistance training [MD=10.90,95%CI:(8.44,13.36),P<0.05],other sports [MD=1.97,95%CI:(1.41,2.54),P<0.05], indoor aerobic[MD=2.43,95%CI:(1.13,3.73),P<0.05],bicycle aerobic[MD=12.18,95%CI:(10.19,14.18),P<0.05],indicating that there was a statistically significant difference between the efficacy of the above four exercise modality interventions in terms of improvement of sperm morphology as compared to the no-exercise group. A two-by-two comparison showed that resistance training was superior to other sports[MD=8.92,95%CI:(6.41,11.46),P<0.05], indoor aerobic[MD=13.35,95%CI:(11.03,15.64),P<0.05], and bicycle aerobic exercise [MD=23.10,95%CI:(19.89,26.22), P<0.05]; other sports were superior to indoor aerobics [MD=4.41,95%CI:(2.99,5.82),P<0.05], bicycle aerobics [MD=14.14,95%CI:(12.21,16.09),P<0.05]; indoor aerobics was superior to bicycle aerobics [MD=9.75,95%CI:(7.42,12.11),P<0.05],and there was no statistically significant difference between the rest of the exercises when comparing the two with each other (P>0.05).

According to the results of the SUCRA probability ranking, resistance training (SUCRA=100.00)>other sports (SUCRA=74.99)>no exercise (SUCRA=49.99)>indoor aerobic (SUCRA=25.01) >bicycle aerobic exercise (SUCRA=0.02), and resistance training is most likely to be the best intervention to improve sperm morphology(See **Figure 5**).

#### 3.5.6 Sperm concentration

A network meta-analysis of the included studies produced 21 two-by-two comparisons. As shown in **Figure 4**, eight studies were reported on sperm concentration with a sample size of 898. The results of the network Meta-analysis showed that there was a statistically significant difference between bicycle aerobic [MD=18.87,95%CI:11.70,25.86,P<0.05], and indoor aerobic[MD=9.53,95% CI:8.97,10.09, P<0.05],both of which were significantly different compared to the no-exercise group.A two-by-two comparison showed that bicycle aerobic exercise was superior to multi-component motion [MD=15.62, 95%CI:(0.34,30.80),P<0.05],competitive sports [MD=18.74, 95%CI:(1.79,35.43), P<0.05], other sports [MD=21.30,95%CI: (14.28,28.27),P<0.05], indoor aerobic[MD=28.40,95%CI:(21.23,35.43),P<0.05]; aerobic endurance exercise was superior to indoor aerobic[MD=17.67, 95%CI:(6.45,29.10), P<0.0]; other sports was superior to indoor aerobic [MD=7.08, 95%CI:(1.88,12.32), P<0.05],and the rest of the differences between the two comparisons were not statistically significant (P>0.05).

According to the results of SUCRA probability ranking, bicycle aerobic (SUCRA=98.32)>aerobic endurance exercise (SUCRA=73.72)> multi-component motion (SUCRA=56.20)>no exercise(SUCRA=45.30)>competitive sports (SUCRA=44.16)>other sports(SUCRA=30.12)>indoor aerobic (SUCRA=2.18),with bicycle aerobic most likely to be the best intervention to improve sperm concentration(see **Figure 5**).

### 3.6 Publication bias

**Figure 6:**
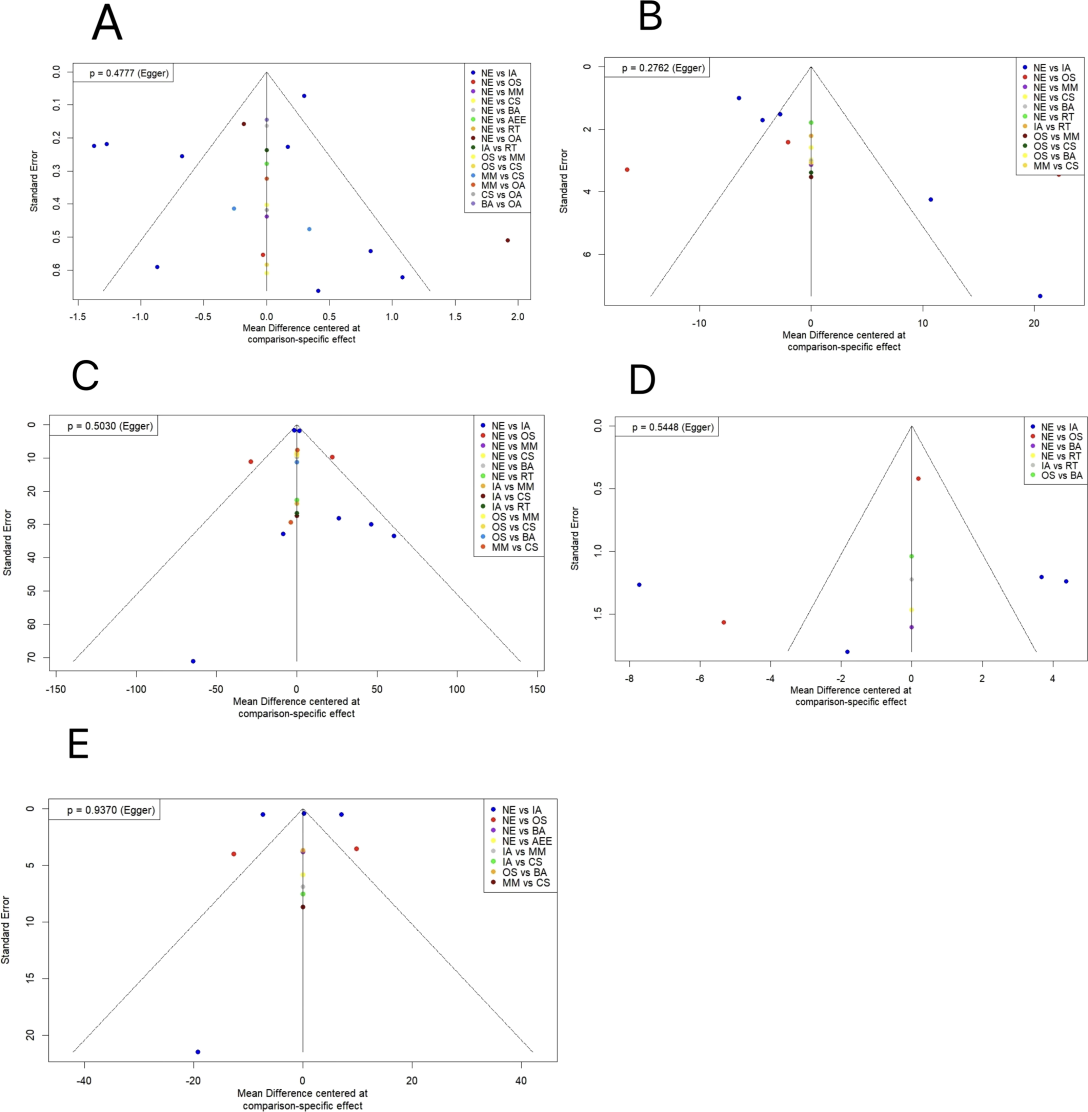
Comparison-correction funnel diagram. **(A)** sperm volume; **(B)** sperm motility; **(C)** total sperm count; **(D)** sperm morphology; **(E)** sperm concentration. OA, Outdoor Aerobic; IA, Indoor Aerobic; MM, Multi-component motion; CS, competitive sports; OS, other sports; RT, resistance training; BA, Bicycle aerobics; AEE, aerobic endurance exercise; NE, No Exercise.

In the included studies, funnel plots were drawn for sperm volume, sperm motility, total sperm count, sperm morphology, and sperm concentration indexes for the publication bias test,and the results showed that the left and right sides of each study point were basically symmetrical, with a P>0.05,indicating that there was no publication bias(see **Figure 6**).

### 3.7 Convergence diagnostics and inconsistency tests

Through the convergence diagnosis of the five outcome indicators, the results show that the PSRF of each outcome indicator is 1, which suggests that the model convergence results are better and the analysis results are more reliable under this model. In addition, the inconsistency test was performed by the node splitting method for direct and indirect comparisons in the network relationship. The results showed that the difference between the direct and indirect comparison results of the two outcome indicators,sperm volume,and total sperm count,was not statistically significant(P>0.05),suggesting that the consistency of the direct and indirect comparison results was good.

## 4 Discussion

### 4.1 Pairwise meta-analysis

The effects of different types of exercise interventions on sperm density, Number of necrotic spermatozoa, and Number of active spermatozoa were explored in Pairwise Meta-analysis. The results showed that in terms of improving sperm density, the effects of indoor aerobic, outdoor aerobic and resistance training were weaker than those of the no-intervention group; for the improvement of Number of necrotic spermatozoa and Number of active spermatozoa, there was no significant difference in the effects of the exercise interventions(P>0.05).

The present study found that different exercise interventions did not produce statistically significant differences (P>0.05) in improving Number of active spermatozoa, which is consistent with previous findings. Some studies have suggested that moderate exercise may positively affect sperm quality by improving blood flow and reducing oxidative stress levels (32), however, excessive or high-intensity exercise may, on the contrary, impair reproductive function and lead to a decrease in the number of active spermatozoa (33).

The present study found that different exercise interventions did not produce statistically significant differences in improving Number of necrotic spermatozoa (P>0.05), and the results of this study are consistent with previous studies. Some related studies have reported that the effect of exercise in promoting male reproductive health may be limited or affected by individual differences, and the improvement of Number of necrotic spermatozoa is not yet obvious or uncertain. Changes in necrotic sperm count may be affected by a variety of factors, such as oxidative stress, sperm apoptosis, etc., and its different forms of exercise may have different effects on these physiological mechanisms, leading to an increase in necrotic sperm count (34, 35).

The present study found that different exercise interventions led to a significant reduction in sperm density in terms of improvement, and the results of this study are consistent with those of previous studies. Maartens et al. (35) showed that outdoor aerobic, especially prolonged high-intensity strenuous exercise, may lead to a reduction in spermatogenesis by affecting the function of the hypothalamic-pituitary-gonadal (HPG) axis and by increasing the level of oxidative stress, which may result in spermatogenesis suppression and decreased reproductive health (36). This suggests that certain high-intensity or specific types of exercise may adversely affect male reproductive function.

### 4.2 Network meta-analysis

The present study used a network Meta-analysis to assess the effects of different exercise interventions on improving multiple aspects of sperm quality. These interventions had varying degrees of effect on important indicators of male fertility, and probability rankings were performed for each indicator. Network Meta-analysis showed that the effects of the various exercise interventions performed differently in terms of improving sperm volume, with outdoor aerobic interventions having the most significant effect, followed by multi-component motion. In terms of improving sperm motility, other sports were the most effective in improving sperm motility. In terms of improving total sperm count, other exercise was the best intervention for improving total sperm count. In terms of improving sperm morphology, resistance training can be a good intervention for improving sperm morphology. In terms of improving sperm concentration, bicycle aerobics was the most effective intervention to improve sperm concentration.

In this study, outdoor aerobic was found to have a significant effect in improving sperm volume in infertile patients. There was a statistically significant relationship between outdoor aerobic, multi-component motion, competitive sports, and indoor aerobic and sperm volume(P<0.05).The result of significant difference between outdoor aerobic and multi-component motion in improving sperm volume is consistent with previous studies. Karaman et al (37) found that moderate-intensity outdoor aerobic was able to prevent deterioration of sperm parameters due to metabolic syndrome by generating a protective response to oxidative damage. Parastesh et al (38) showed that endurance training, resistance training, and concurrent training significantly increased serum testosterone and LH levels, which further improved sperm parameters. In addition, a significant effect of competitive sports on sperm volume is inconsistent with previous studies, Vaamonde et al. (39) showed that competitive sports have a positive effect on overall reproductive health, and that professional athletes undergoing long-term high-intensity, high-load competitive sports caused testicular damage as well as hormonal alterations, leading to increased levels of oxidative stress in seminal plasma and decreased enzyme antioxidant defenses, which caused spermatogenesis to be impaired, thus reducing sperm quality and fertility potential (40). It may be due to the difference in the scope of the study population with the results of the present study, compared to the present study, which utilized indirect comparative data to expand the scope of the study population to a certain extent, and the results may be more stable. A significant difference in sperm volume with indoor aerobic exercise is consistent with previous studies, Fahaid et al. (41) found that moderate indoor aerobic can enhance the antioxidant capacity of the testes and protect sperm cells from oxidative DNA damage by up-regulating the antioxidant enzymes and glutathione content.

In this study, we found that other sports had a significant effect on improving sperm motility in infertile individuals. There was a statistically significant relationship between other sports, indoor aerobic, and resistance training and sperm motility (P < 0.05), a result that is consistent with the positive effects of whole body exercise and diversified exercise on improving sperm motility in some previous studies (42). Bisht et al (43) showed that infertility patients improved semen parameters through yoga exercise, which is known to improve reproductive health and fertility by triggering neurohormonal mechanisms, reducing stress and anxiety, and improving autonomic function (44). However, Bahadorani et al. (45) reported that moderate-intensity indoor aerobic exercise activated the production of antioxidants, which reduced oxidative damage in testicular tissues and enhanced sperm DNA integrity. In addition, Maleki BH et al. (46) 2018 found that moderate-intensity exercise and resistance training interventions further improved sperm viability by activating superoxide dismutase which in turn inhibited oxidative stress processes (47), reduced lipid peroxidation levels (48), and thereby ensured spermatogonial cellular integrity (49), in obese German men with sedentary behaviors over a 24-week period of incremental loading resistance training. These exercise interventions were shown to have an important role in the improvement of reproductive health, especially the other sports and indoor aerobic, which showed more prominent efficacy in several comparisons.

In this study, we found that other exercises had a significant effect on improving sperm count in infertile patients. There was a statistically significant relationship between other exercises and total sperm count (P < 0.05), and the results of this study are consistent with those of previous studies. Existing relevant studies have shown that exercise can improve male reproductive health through a variety of mechanisms, including regulating hormone levels, improving blood circulation, and reducing body weight (50). The effects of other exercises may be related to their higher energy expenditure and more comprehensive physical activity patterns, e.g., yoga, Pilates, etc., which can help to elevate the levels of reproductive hormones and thus promote sperm production. In addition, Bhat et al. (51) showed that yoga, as a complementary and alternative medicine modality, activates neurohormonal pathways, and yoga poses promote blood circulation, strengthen pelvic and perineal muscles, increase contractions, and enhance autonomic functioning, ultimately enhancing reproductive health.

The present study found that resistance training had a significant effect on improving sperm morphology in infertile individuals. It suggests that exercise intervention has a potential positive effect in promoting normalization of sperm morphology, especially in resistance training. There was a statistically significant relationship between resistance training, other sports and sperm morphology (P < 0.05), and the results of this study are consistent with previous studies. Related studies have shown (52) that resistance training can enhance reproductive function by boosting testosterone levels and improving body composition, a result that emphasizes the potential benefits of different types of exercise on male reproductive health, especially the superiority of resistance training (53). Yadav et al. (54) showed that yoga enhances antioxidant capacity in the body and reduces sperm damage caused by oxidative stress; it regulates hormones by reducing stress and lowering cortisol levels and regulating hormonal balance, which in turn promotes sperm health. Yoga helps to improve the quality of sperm morphology through overall health improvement and provides positive support for male reproductive health.

In this study, we found that bicycle aerobics had a significant effect on improving sperm concentration in infertile individuals, and there was a statistically significant relationship between bicycle aerobics and sperm concentration (P < 0.05), which was inconsistent with the results of previous studies. A related study (55) showed that continuous cycling can also affect the scrotum and cause a decrease in sperm count, due to the slightly improper cycling posture, duration and frequency can cause damage to male reproductive health. There may be a difference with the results of the present study due to factors such as the intervention time and frequency of the study participants. In contrast, the present study utilized appropriate exercise intensity and frequency and a systematic intervention design, which to some extent provided stability and comparability of the results. In conclusion, exercise can affect reproductive health by improving body composition, enhancing blood circulation and regulating endocrine levels (56, 57). The superior effect of bicycle aerobic may be related to its high-intensity and sustained nature, which can effectively enhance cardiovascular health and metabolic function, thus promoting sperm quality (58).

Moderate exercise has a positive impact on sperm quality. Studies have shown that moderate-intensity exercise (e.g., 30-60 minutes of aerobic exercise 3-4 times per week) significantly improves sperm concentration, viability and morphology. Specifically, 150-300 minutes of moderate-intensity exercise per week increased sperm concentration by 12-17% and viability by 8-10%. However, high-intensity exercise (e.g., >10 hours of vigorous exercise per week) may reduce sperm quality and decrease sperm concentration by 14-20%. In terms of frequency of exercise, moderate intensity exercise 3-5 times per week is most effective, whereas daily high intensity exercise may damage sperm DNA integrity due to increased oxidative stress. In terms of duration, moderate-intensity exercise interventions lasting 3-6 months significantly improved sperm parameters, but high-intensity training for more than 1 year may result in decreased sperm quality. Therefore, a moderate-intensity exercise programme of 30-60 minutes 3-5 times per week is recommended for optimal reproductive health benefits.

The effect of exercise on male infertility is mainly through the regulation of hormone levels, oxidative stress, testicular function and psychological state. Firstly, moderate exercise promotes spermatogenesis and maturation by regulating the hypothalamic-pituitary-gonadal (HPG) axis to increase testosterone and luteinising hormone (LH) levels, and increases follicle stimulating hormone (FSH) secretion to optimize spermatogenesis. However, high-intensity exercise may inhibit HPG axis function due to elevated cortisol and interfere with testosterone secretion. Secondly, moderate exercise activates antioxidant enzymes (e.g., superoxide dismutase, glutathione peroxidase), reduces reactive oxygen species (ROS) damage to sperm DNA, and maintains sperm integrity, whereas excessive exercise exacerbates oxidative stress, leading to testicular tissue damage and sperm apoptosis. In addition, exercise enhances the efficiency of spermatogenesis by improving the local blood flow and metabolic environment in the testes, regulates antioxidant mechanisms to protect sperm cells, and reduces the release of inflammatory factors to optimize the reproductive microenvironment. Finally, in terms of psychological regulation, exercises such as yoga and Pilates can reduce cortisol levels and improve autonomic function, while aerobic exercise can indirectly improve fertility by promoting the release of endorphins to relieve psychological stress. Overall, moderate and scientific exercise interventions are valuable in improving male infertility, but the possible negative effects of high-intensity exercise should be avoided.

## 5 Strengths and limitations

The results of this study should be interpreted with its limitations in mind. Firstly, the language of the literature included in this study was limited to Chinese and English, and there may be publication bias. Second, only a few of the included studies reported allocation concealment and blinding, which may have some selection bias. Third, there were significant differences in the effects of different exercise interventions on sperm quality in this study, and due to insufficient standardization of exercise intensity, frequency and time, and small sample sizes of some indicators, there may be a certain bias in the comparison of related indicators and other results, making it difficult to draw consistent conclusions; fourth, the countries, medical conditions, ages, BMIs, exercise intervention regimens, and testing criteria and methods could not be rationally categorized.

Due to the limitation of the quality and number of included studies, different exercises, testing standards, and methods may have different effects on the results of these physiological mechanisms, so more high-quality studies and recognized testing standards are needed in the future to further elucidate the optimal exercise interventions. The present study used bias analysis to obtain a lower bias situation, which makes it possible to improve the reliability of the results and the credibility of the conclusions, enhance the generalizability of the results, and make it possible to provide more solid evidence for clinical decision-making; in addition, the use of funnel plots can identify potential sources of bias, which ultimately helps to increase the academic value and the potential of application of the present study on the role of different exercises in improving spermatozoa, and promotes the robustness and repeatability of the study’s conclusions. The fact that different exercise interventions have significant advantages for improving sperm quality, and that exercise may be a safe activity and more cost-effective than other treatment strategies for male factor infertility, could help to promote such interventions in clinical or public health settings in the future.

## 6 Conclusions

In summary, this study provides the most extensive analysis to date by summarizing the effects of different exercise interventions on sperm quality. The results of the study showed that outdoor aerobic had a significant effect on improving sperm volume in infertile patients; other sports had a significant effect on improving sperm motility and total sperm count in infertile individuals; resistance training had a significant effect on improving sperm morphology in infertile individuals, and bicycle aerobic had a significant effect on improving sperm concentration in infertile individuals (59).

## Data Availability

The original contributions presented in the study are included in the article/supplementary material. Further inquiries can be directed to the corresponding author.
